# Fracture resistance of endodontically treated teeth with different direct corono-radicular restoration methods

**DOI:** 10.4317/jced.53160

**Published:** 2017-03-01

**Authors:** Horieh Moosavi, Safora Afshari, Fatemeh Manari

**Affiliations:** 1 Associate Professor of Dental Materials Research Center and Department of Operative Dentistry, Faculty of Dentistry, Mashhad University of Medical Sciences, Mashhad, Iran; 2 Assistant Professor of Dental Materials Research Center and Department of Operative Dentistry, Faculty of Dentistry, Mashhad University of Medical Sciences, Mashhad, Iran; 3 DDS, Dentist, Mashhad University of Medical Sciences, Mashhad, Iran

## Abstract

**Background:**

Endodontically treated teeth are widely considered to be more susceptible to fracture than vital teeth. Obturation procedures and post placement have been a main cause of vertical root fracture.

**Material and Methods:**

Forty-eight human premolars with standardized weakened roots were endodontically treated and allocated to four experimental groups (n=12). After root canal treatment, in group 1, fiber posts #1 were cemented in root canals using Estelite Core Quick, and the crowns were restored with resin composite. For group 2 and 3, the roots and crowns were restored using a light-cured and self-cured adhesive and resin composites respectively. In group 4, it was used the Panavia F 2.0 resin cement and resin composite for corono-radicular reconstruction. In group 5, the teeth remained untouched. After 24 hours storage and 1000 thermocycles, samples were loaded at a cross head speed of 1 mm per minute.

**Results:**

A significant difference was observed in fracture resistance among groups 4 and 5 compared to other groups.

**Conclusions:**

Root reconstruction with fiber post and Panavia resin cement, and crown building using light-cured resin composite resulted in increased fracture resistance equal to that of intact teeth.

** Key words:**Fracture resistance, fiber post, resin cement, resin composite.

## Introduction

Endodontically treated teeth (ETT) are potentially weaker than vital teeth against chewing forces and may fracture more easily. For many years, post and core systems have been used as foundational materials for final restoration of ETT that have lost most of their coronal tooth structure. Posts and cores can be custom-made or prefabricated (1,2). In the early 1990s, prefabricated, finally polymerized fiber-reinforced composite (FRC) root canal posts were introduced to the market. FRC posts have been suggested to have certain advantages over metal posts (3). The elasticity modulus of an FRC post is closer to that of dentin when compared with rigid metal posts. Lower stress concentrations are therefore transmitted to the root, diminishing the risk of root fractures (4-6). However, FRC root canal posts have been criticized on grounds of their flexural properties as well as for undesirable adhesion to luting cements and core build-up composites (3,4). On the other hand, many investigators have suggested that these materials boast the advantage of reducing the risk of root fracture thanks to their modulus of elasticity (16-40 GPa) being comparable with that of composite resins (5.7-25 GPa) and dentin (18.6 GPa) (7,8). Despite these advantages, bonding to radicular dentin offers less favorable conditions than coronal dentin, and thus it is still considered the frailest bond in terms of restoration (3). The success of the root-dentin adhesive-restorative system is directly linked with the hybridization quality created through the infiltration of adhesive system into the demineralized dentin substrate (9). In addition, the distribution of resin cement in the post space during the luting procedure, besides the anatomical and histological characteristics of the root dentin contribute significantly to the bond strength between the resin luting agent and root canal regions (3). Adequate polymerization of the luting agent is necessary to achieving high mechanical properties of the resin cement and, in turn, to obtaining an adequate bond quality to the root canal walls. However, since light intensity declines with the increase in the distance from the light source tip in light polymerization systems (5), the apical areas of post preparation in the root canal continue to pose a challenge in terms of the bonding protocol. Consequently, there exist additional difficulties with regard to the insertion and light-curing of adhesive restorative systems. In light of this fact, one may hypothesize that materials which do not rely merely on light activation might produce better retention in the apical thirds of root canals (6). A variety of luting agents weather light, dual or self-cure and corresponding adhesive systems have been proposed for bonding FRC posts to root canal dentin. Recently, some self-adhesive resin cement which has a dual-cure mechanism and requires no dentin pretreatment has been introduced on the dental market (10). The null hypotheses were that the various corono-radicular reconstruction of fracture resistance are not significantly influenced by 1) type of resin composite or 2) cement resin and 3) there was similar mode of failure.

## Material and Methods

Sixty freshly sound human premolars had been extracted for orthodontic reasons were gathered following informed consent approved by the Commission for Medical Ethics of the University of Medical Sciences (N#910165). Teeth randomly partitioned into five groups (n=12). Remained soft tissue, calculus and plaque removed with rubber cap and slurry of pumice after hand scaling instrument, then stored in 0.1% thymol solution until operation time. In 48 teeth, the crowns were cut from 3 mm above the CEJ and mesiodistally cavities were prepared, measuring 3 mm buccolingually dimension. Upon completion of root canal treatment, the following procedures were followed: in the first group, fiber posts #1 (Tokuyama Dental Corp.,Tokyo, Japan) with length of approximately 8 mm were cemented in root canals applying Estelite Core Quick (Tokuyama Dental Corp.,Tokyo, Japan) as the manufacturers’ instructions, and the crowns were restored with resin composite; Estelite Sigma Quick (Tokuyama Dental Corp.,Tokyo, Japan). Concerning the second group, the roots and crowns were restored using a combination of self-etch adhesive; Bond Force (Tokuyama Dental Corp.,Tokyo, Japan) as the manufacturers’ instructions, and light-cure resin composite; Estelite Sigma Quick (Tokuyama) that was packed incrementally with plugger and condenser from apical to coronal of preparations. For the group 3, self-cured composite; Master Dent (USA), and the corresponding adhesive in the package were used to reconstruct the roots and crowns similar to group 2. With respect to the group 4, the self-etch resin cement; Panavia F 2.0 (Kuraray, Dental Inc., Okayama, Japan) was used for cementation of fiber posts and the crown building was performed employing resin composite; Clearfil AP-X (Kuraray, Dental Inc., Okayama, Japan). Regarding the fifth group (control group), the teeth remained untouched. All materials, compositions and procedures used in the study were displayed in  Table 1. Samples were stored in distilled water at 37°C for 24 hours after that were thermocycled (5-55°C, 1000 cycle 60-second dwelling time and 30 second transfer time) by an automatic thermocycler (Lab co. Mashhad, Iran). All procedures were performed by a single operator. Fracture resistance was tested as described by Mondelli *et. al* (11). The teeth were mounted in a customized fixture and subjected to axial compressive loading with crosshead speed of 1.0 mm min-1 (Santam Instron, Tehran, Ir). The vertical loading force was applied through an 8 mm-diameter stainless steel ball parallel to the tooth axis. The contact points were approximately half way up the cusp triangular ridge. Fracture resistance was recorded at the peak of the load-displacement curve. In addition, the fracture patterns were recorded using a simplified classification of cracked tooth syndrome proposed by the American Association of Endodontics (12). The fracture patterns recorded were type 1 fracture – fractured cusp – which may extend to the cervical third of the crown or root (restorable) and type 2 fracture – fractured tooth – which includes cracked tooth and split tooth (nonrestorable). Fracture resistance of premolars between the five groups was compared using the ANOVA/ Tukey’s test. Fracture patterns of the five groups were analyzed with the Fisher’s Exact tests. Pairwise comparison was carried out to calculate the odds ratio. The significance level was set at 0.05.

**Table 1:**
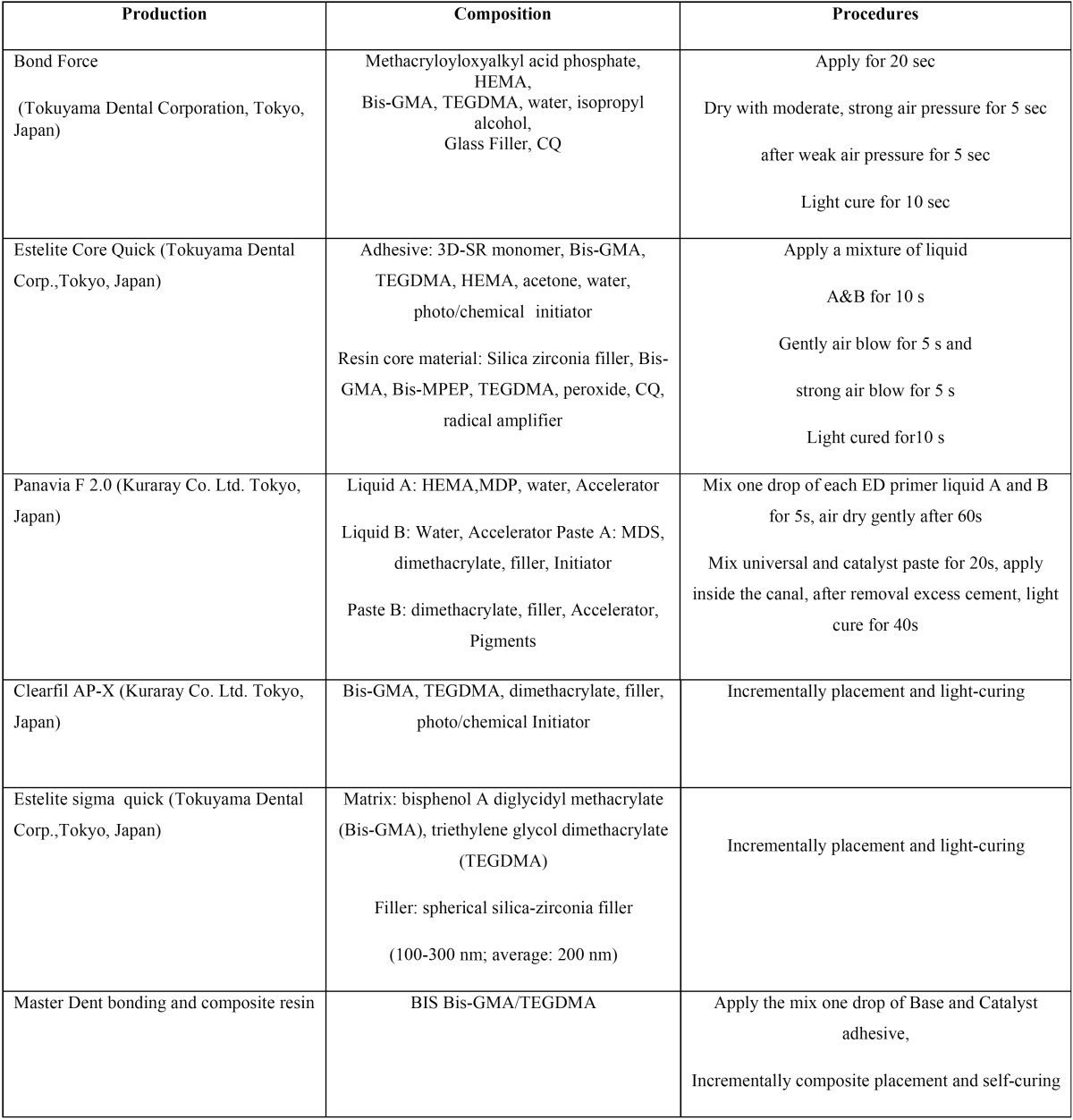
Names, manufacturers, compositions and procedures of the products used in the present study.

## Results

**Figure 1:**
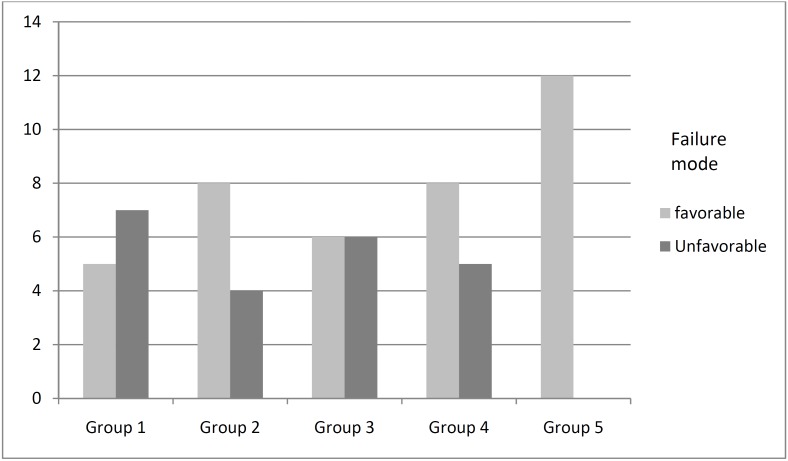
The frequency of the failure mode in the experimental groups.

The maximum and minimum fracture resistance was seen in the fourth and third groups respectively ( Table 2). Findings indicated a significant difference in the mean values of fracture resistance among experimental groups (*P* < 0.05). A significant difference was observed in the mean values of fracture resistance among groups 4 and 5, in comparison with the other groups (*P* < 0.05), while there was no significant variation regarding fracture resistance among groups 1, 2, and 3 (*P* > 0.05). Tukey’s test pointed out significant differences between the groups in terms of the mean fracture resistance, between groups 4 and 5, compared with other experimental groups (*p* < 0.05). No significant difference could be seen in fracture strength between groups 1, 2 and 3 (*p* > 0.05) ( Table 3). The frequency of failure mode was determined for all experimental groups in figure 1.

**Table 2:**
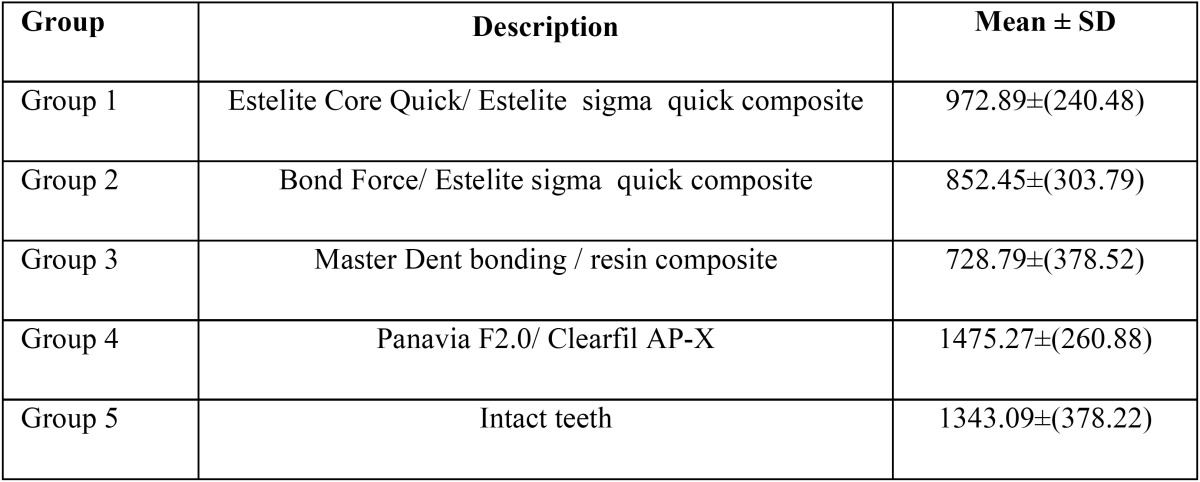
Mean and standard deviation of fracture resistance in experimental groups.

**Table 3:**
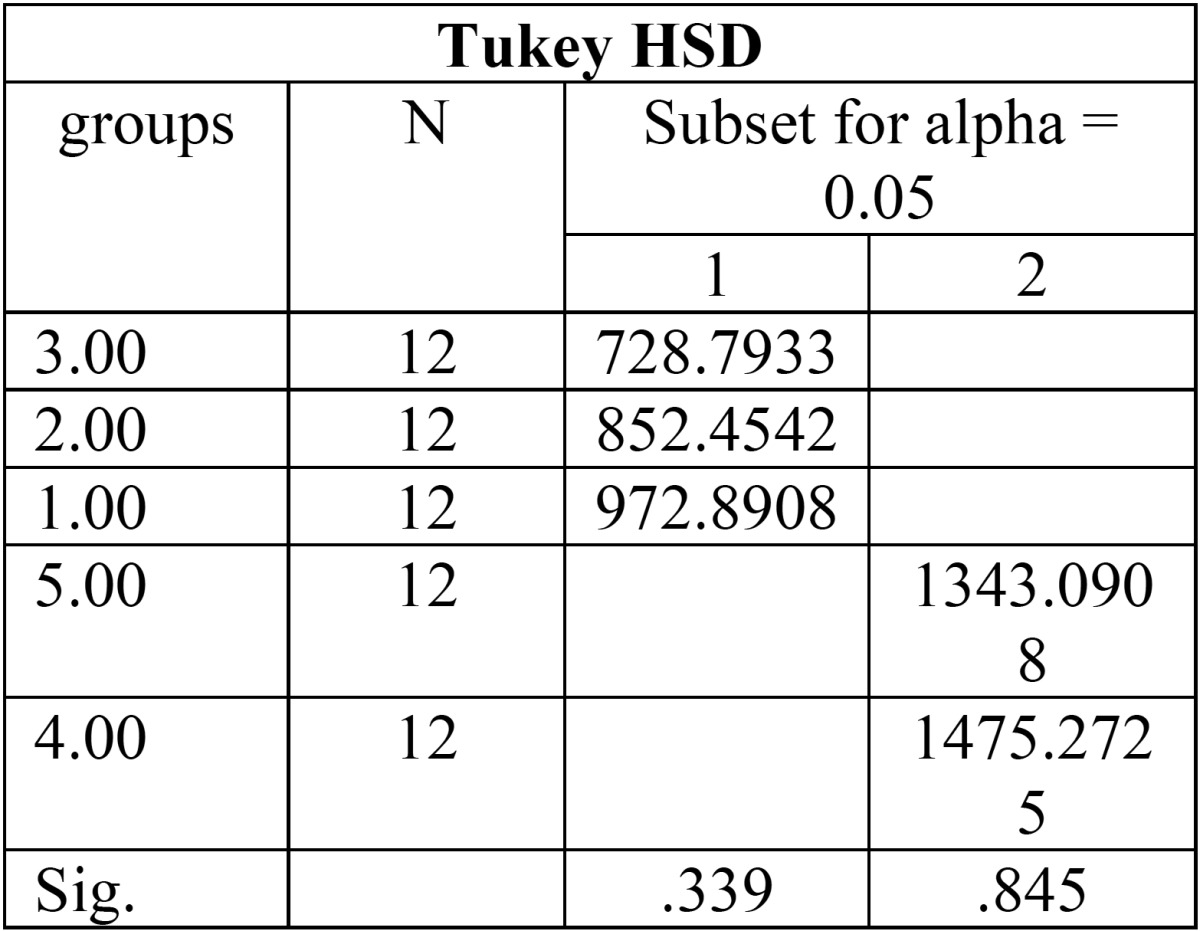
Comparison between two experimental groups by Tukey test.

The number of desirable failures in experimental groups 2 and 4 outnumbered the undesirable occurrences. In group 1, the undesirable failure rate was higher than favorable failure. Finally, in group 3, the rate of both failure types were the same. Fisher’s Exact test showed a significant difference in the mode of failure (*P* = 0.019). Considering all experimental groups, the total number of desirable failures was 38 and that of undesirable failures 22.

## Discussion

Management of endodontically treated tooth (ETT) with severe destruction of crown’s buildings has always been an challengeable issue. To repair these teeth, patients and dentists have invariably been looking for a method with greater stability and survival rate, one which does not impose high costs or complicated procedures (13). Nowadays, most dentists tend to utilize prefabricated posts due to their functional, cost-effective, and conservative properties (14). In the process of this commonly-practiced treatment, a massive amount of gutta-percha is fitted to create the right space for the post and to make possible certain accessory canals. Thus sealing using post, core, and adhesive materials becomes a mandatory part of the treatment (15). Composite cores together with dowels of cemented tooth-colored posts are typically used to restore endodontically treated tooth (16). One of the main causes of failure in endodontic treatment of teeth with extensive damage is reduced fracture resistance both in the teeth and the restoration. The aim of the present study was to evaluate fracture strength of endodontically treated premolars with different direct corono-radicular restoration methods so we did not use indirect crown restorations. In this study, there were significant differences in the mean of fracture resistance among groups 4 and 5 in comparison to other experimental groups, while no significant differences were observed between groups 1, 2 and 3. One of the main challenges is always the bonding between the post and the cement. Among the influencing factors are the solvent in the adhesive as well as the rate and the power of evaporation. Ethanol and acetone are commonly used in one-step self-etching adhesives as organic solvents mixing water with hydrophobic components (5,9). Adhesives applied in this study, with the exception of the third group, were single-step self-etching. Before polymerization of the optical adhesive, removal of the solvent/water from single-step self-etching adhesive is recommended to achieve optimal adhesive polymerization. In case the water/solvent is partially removed, it can become trapped in some layer of the adhesive, jeopardizing the mechanical properties, which can consequently reduce the amount of polymerization and lead to subsequent compromise the bond strength (5,17). Therefore, it is likely that the residual solvent/water in the adhesive layer was a factor reducing the fracture resistance of the teeth in the first and second groups of our study. Full curing of adhesive in restorations has been one of the success factors (6,17). In the first and second groups, following manufacturers’ recommendations, light curing adhesive was employed. Yet, insufficient polymerization occurred possibly due to lack of adequate light focus onto the apical zone. However, in the fourth group, in which dual-light-curing mechanism was the choice, more complete polymerization ensued, and therefore distinguished fracture resistance was obtained. Usually, chemical polymerization delivers less than the light type (17). Thus, the third group of the study, having experienced chemical polymerization, showed the least degree of resistance to failure. It seems that using resin composite instead of cemented posts deleted one of the bonded interfaces, and therefore the bonding relied only on adequacy of bond strength between the tooth / adhesive / and composite. Nevertheless, the problem of encountering high c-factor during cementation was removed when this method was applied (18). In the second and third groups of the study, composite pins were used instead of transparent posts. Generally, under condensation polymerization shrinkage stresses caused by shrinkage of composite resin can be a fundamental setback affecting the bonding to dentin (17). Shrinkage stress could impact dentin bonding and reduce the bond strength, which could mean failure in the dentin / resin interface. In order to deal with composite resin shrinkage stress, one must ensure optimum mechanical properties of the adhesive before starting the polymerization of the composite resin. Lower fracture resistance observed in the third group indicated insufficient chemical polymerization of adhesive, and ultimately the inability of the adhesive to cope with the composite resin’s shrinkage stress in cavities with high C-factor, which eventually resulted in decreased resistance to fracture. Another reason for lower fracture resistance seen in the second and third groups compared with other groups might have been lower density; specifically the lower density of the composite pins versus prefabricated posts, and possibly of the adhesive-dentin hybridization structure. In order to apply composites in root canals, the layer technique was used, which could explain the reduction of the fracture resistance in these samples. In total, the third group of the study, probably due to the use of chemical curing and composite pins, revealed the lowest level of fracture resistance value. On the contrary, the fourth group, possibly thanks to: the use of single-step self-etching adhesives; as well as adopting a curing method using dual-curing mechanism paired with light radiation; and also the application of transparent pins within the root canals, yielded the highest fracture resistance values similar to intact teeth (control group). Another influence factor for the higher fracture resistance of the fourth group compared with the first three groups could have been the composite core build-up material (19). It is likely that the elasticity modulus of Clearfil AP-X composite is greater than that of core build-up composites used in other experimental groups. Similar to a previous study, Reforpin can be used as an alternative to resin composite for internal reinforcement of weakened roots (20). The current study, unlike Prisco’s study (21), which suggests there is a significant difference between adhesion properties of different types of cement-post systems, showed a significant difference in fracture resistance of two resin cements, namely Panavia and Estelite Core Quick Resin Cement. This finding may be related to the fracture resistance level of these two resin cements. Also, it could also be associated with the differences in usage, their constituent cements, and their resin composite core build-up. In severely damaged roots, a fiber post connected with adhesive may improve fracture strength and ensure better stress distribution and transmission, plus strengthening the teeth (13,14). Since application of adhesive cements presents certain advantages, such as lower leakage and better retention, they are presumably a preferred option compared to metallic posts (22). Resin cements bind chemically and micromechanically to post and dentin such that has not been observed in other types of cement (23). The third hypothesis did not prove; there was significant difference in the mode of failure in experimental groups. However, the four experiments performed, which revealed the total number of favorable failures as 26 and the unfavorable as 22, increased hopes of achieving greater resilience for endodontically treated teeth healed with tooth-colored materials. A previuos study was in consistent with the findings of this study, that employing composite resins together with lateral fiber glass posts seem to be an effective method for improving the biomechanical behavior in widened roots (24). Makade concluded that teeth restored with fiber glass posts showed the easiest-to-restore type of failure (19). Somewhat similar to this study, Sorrentino’s study exhibited samples restored with posts, which showed mainly repairable fracture, while teeth restored without posts showed mostly irreparable failure (25). Overall, it seems that loss of hard tissue due to root canal treatment have to be considered responsible for the increased fracture risk of ETT especially unfavorable fracture (26,27). The authors recommend further laboratory studies examining bond strength and leakage using other materials. Likewise, more clinical trials in the following years may provide more valid results.

## Conclusions

In case of endodontically treated teeth with mass destruction, applying clear posts and using the resin cement Panavia for root reconstruction, plus employing light-cure resin composite for rebuilding of the crowns increased their fracture resistance to the same level as healthy teeth.
